# Isolation and identification of mosquito biting deterrents from the North American mosquito repelling folk remedy plant, *Matricaria discoidea* DC.

**DOI:** 10.1371/journal.pone.0206594

**Published:** 2018-10-31

**Authors:** Charles L. Cantrell, Abbas Ali, A. Maxwell P. Jones

**Affiliations:** 1 Natural Products Utilization Research Unit, United States Department of Agriculture, Agricultural Research Service, University, Mississippi, United States of America; 2 National Center for Natural Products Research, The University of Mississippi, University, Mississippi, United States of America; 3 Gosling Research Institute for Plant Preservation, Department of Plant Agriculture, University of Guelph, Guelph, Ontario, Canada; National Taiwan Ocean University, TAIWAN

## Abstract

*Matricaria discoidea* DC. (Asteraceae) has documented use as an insect repellent by Blackfoot Indians and other indigenous groups of North America. This investigation was conducted to evaluate this practice and systematically identify chemical constituents responsible for any insect repelling effect by utilizing a mosquito (*Aedes aegypti* (Linnaeus)) biting deterrent bioactivity-directed purification approach. Hydrodistilled oil from dried aerial parts of *M*. *discoidea* was the most bioactive crude extract generated and was as. Fractionation of this extract, followed by re-evaluation for mosquito biting deterrence using the K & D (Klun and Debboun) bioassay, produced many active fractions that were subsequently evaluated by spectroscopic techniques and the most active compounds were determined to be α-terpineol, spathulenol, and neryl isovalerate. A & K (Ali and Khan) large cage in vitro evaluation of pure compounds isolated from *M*. *discoidea* indicated α-terpineol to be the most active compound providing complete protection at 25 μg/cm^2^. This is the first report on the mosquito repellency of neryl isovalerate and scientific evidence reported here validates the traditional use of *M*. *discoidea* as a biting-insect deterrent.

## Introduction

As defined by the Environmental Protection Agency (EPA), biopesticides include naturally occurring substances that control pests (biochemical pesticides), microorganisms that control pests (microbial pesticides), and pesticidal substances produced by plants containing added genetic material (plant-incorporated protectants). Natural products from plants have been extensively used in the discovery of new biochemical pesticides with more success seen with bioinsecticide discovery programs as compared with biofungicide and bioherbicides [1].

Like many biopesticide discovery research programs, mosquito repellent research programs must also identify sources of lead natural compounds and biochemical structures for development or optimization. Biopesticide discovery programs utilize a range of proven techniques for the identification of natural compounds such as random screening of plants or microbial extracts, targeting of extracts from particular families known to possess these traits, targeting of certain compound classes, and screening of available crude extract or pure compound repositories and libraries [1]. All of these techniques seem to rely on the ability to perform high-throughput screening to streamline the discovery process and it is common practice to assemble such repositories and libraries into a 96 well-plate format while evaluating sub milligram quantities of tested preparations. Mosquito repellent discovery offers unique challenges that are not conducive to such high-throughput programs forcing us to look more carefully at alternative methods for discovery of novel biochemicals. Examination of traditional or “folk” remedies used by native peoples has proven to be a valuable source of information when selecting plants to investigate for the presence of insect repelling natural compounds [2–5].

An example of a traditional repellent remedy investigation that originated from knowledge communicated from a grandfather to a grandson pertains to using leaves from the American beautyberry plant, *Callicarpa americana* L., to prevent insect bites. A systematic investigation using a bioassay-guided fractionation approach led to the identification of spathulenol, intermedeol, and callicarpenal. Callicarpenal, intermedeol, and spathulenol proved to be highly effective biting deterrents against the mosquito species *Anopheles stephensi* and *A*. *aegypti* [2]. Another example can be found with Sweetgrass, *Hierochloe odorata* (L.) P. Beauv., where Flatheads of Montana and Blackfoot of Alberta would use braided plant material in a sachet in clothing or burn them from one end as an insect repellent. A bioassay-guided fractionation approach supported the traditional use of this plant and identified phytol and coumarin as the primary biting deterrent compounds [5].

Two additional traditional folk remedy examples from separate continents were burned and used as spatial insect repellents. Both were investigated independently and led to the same class of compounds responsible for the activity, namely fatty acids [3–4]. One of these, *Jatropha curcas* L., utilized the seed pressed oil that was burned in an oil lamp with the function of repelling biting insects. The other, breadfruit or *Artocarpus altilis* (Parkinson) Fosberg, utilized the male inflorescence which was also burned and functioned to prevent insect bites. The primary mosquito deterrent constituents from *A*. *altilis* were determined to be capric, undecanoic, and lauric acids while those from *J*. *curcas* were oleic, palmitic, linoleic, and stearic acids in addition to various triglycerides.

Pineapple weed, *Matricaria discoidea* DC. (Asteraceae), has been reported to have been used by various indigenous peoples of North America for medicinal purposes, perfume, and as an insect repellent [6–9]. However, no reports were found on the systematic investigation of *M*. *discoidea* for its mosquito repelling constituents. Described in detail below is the *Aedes aegypti* bite-deterring bioassay-guided fractionation of *M*. *discoidea*. A total of eight isolated compounds with biological activities against *A*. *aegypti* are reported. *A*. *aegypti* was chosen as the target mosquito species as it is a known vector of several important viruses including yellow fever, dengue, chikungunya, and Zika.

## Material and methods

### Plant material

Wild *Matricaria discoidea* plant material was collected from the University of Guelph Campus during July, 2014. Plants were in flower and the whole areal portion was collected. The fresh plant materials were placed in mesh bags and dried under forced air at approximately 40°C for 2–3 days or until fully dry. A voucher specimen was pressed and deposited in the University of Guelph Herbarium, accession number 100804.

### Gas Chromatography-Mass Spectrometry-Flame Ionization Detection analysis

As described in detail previously [10], essential oil samples and isolated compounds were analyzed by GC-MS-FID on an Agilent 7890A GC system equipped with an Agilent 5975C inert XL MSD with triple axis detector and an Agilent 7693 autosampler.

### Retention Index and Kovat Index analysis

Retention Index and Kovat Index analysis [11] were performed on all purified compounds and the mass spectra were compared with the NIST mass spectra database. Commercial standards were purchased for putatively identified constituents and analyzed to provide unequivocal identification. Commercial standards α-terpineol, D-limonene, and β-farnesene were obtained from Sigma-Aldrich (St. Louis, MO) and were injected and compared with retention time and mass spectra data of purified compounds for identification.

### NMR spectroscopic analysis

^1^H, ^13^C, distortionless enhancement by polarization transfer (DEPT), heteronuclear single quantum coherence (HSQC), and heteronuclear multiple bond correlation (HMBC) NMR spectra were recorded in CDCl_3_ on a Bruker 400 MHz spectrometer (Billerica, MA, USA).

### Preparation of crude extracts

*M*. *discoidea* aerial parts (21 g) were air-dried followed by grinding for 5 min in a Retsch PM 400 ball grinder. Ground plant material was extracted at room temperature for 24 hours using 250 mL of hexane, providing 490 mg of extractables after evaporation of the solvent. Dried marc was subsequently extracted using 250 mL of methylene chloride (DCM), providing 272 mg of extractables following evaporation of the solvents. This process was again repeated using 250 mL of ethanol (95%) as the extraction solvent, providing 902 mg of extract.

Hydrodistillation was accomplished by placing air dried *M*. *discoidea* leaves into a 5 L round bottom flask followed by the addition of 2.5 L of deionized water. Dried leaves were subjected to hydrodistillation for 24 h. Hydrodistillation was performed using a Clevenger-type apparatus containing 3 mL of n-*pentane*. The organic layer from the distillations was combined and dried under a stream of dry nitrogen, resulting in a yield of 171 mg of essential oil. This process was repeated using multiple batches of plant material.

### Fractionation of the essential oil

Column chromatography was performed on a Biotage, Inc. (Charlotte, NC) IsoleraTM pump equipped with an IsoleraTM flash collector and variable wavelength detector. The essential oil (299 mg) was fractionated using an XP-Sil, 100 g, SNAP cartridge (40–63 μm, 60 Å, 40 x 150 mm) running at 50 mL min^-1^ using a two solvent gradient from hexane to hexane:ethyl acetate (70:30) over 2400 mL while monitoring at 254 nm. Column eluate was collected into 22 mL portions and based on TLC similarities, recombined into 10 fractions (A, 1–12, 4 mg; B, 13–24, 40 mg; C, 25–27, 68 mg; D, 28–29, 10 mg; E, 30–33, 7 mg; F, 34–39, 15 mg; G, 40–44, 32 mg; H, 45–50, 22 mg; I, 51–57, 14 mg; J, 58–80, 76 mg). This process was repeated twice using multiple batches of essential oil from above.

Fractions A, B, and I were identified as D-limonene, β-farnesene, and α-terpineol, respectively, using RI and KI data as well as comparison of retention time and MS data from commercially available standards.

Subfractionation of fraction C (68 mg) was accomplished using an XP-Sil, 100 g, SNAP cartridge (40–63 μm, 60 Å, 40 x 150 mm) running at 40 mL min^-1^ using a two step linear gradient from 100% hexane to 50% hexane:ethyl acetate (90:10) over 1600 mL followed by a step from 50% hexane to 100% hexane:ethyl acetate (90:10) over 600 mL. Column eluate was collected into 22 mL portions and based on TLC similarities, recombined into 5 fractions (C-A, 43–50, 5 mg; C-B, 51–57, 59 mg; C-C, 58–65, 5 mg; C-D, 66–70, 5 mg; C-E, 71–88, 4 mg).

Subfraction C-A was identified as dendrolasin based on comparison of ^13^C NMR spectral data with that reported in the literature [12]. ^13^C NMR (101 MHz, C_6_D_6_) δ 142.9, 139.3, 135.7, 131.2, 125.2, 124.9, 124.4, 111.4, 40.2, 28.9, 27.1, 25.9, 25.4, 17.8, 16.1.

Subfraction C-B was identified as neryl isovalerate based on comparison of ^1^H NMR spectral data with that reported in the literature [13]. ^1^H NMR (400 MHz, CDCl_3_) δ 5.30 (m, 1H), 5.08 (m, 1H), 4.57 (d, J = 7.2 Hz, 2H), 1.69 (s, 3H), 1.66 (s, 3H), 1.58 (s, 3H), 0.96 (d, J = 7.2 Hz, 6H). ^13^C NMR (101 MHz, CDCl_3_) δ 173.2, 142.2, 131.8, 123.9, 118.6, 61.1, 43.6, 39.6, 26.4, 25.9, 25.8, 22.5, 17.8, 16.5.

Fraction D was identified as isohumbertiols, specifically isohumbertiol B and isohumbertiol D in a 1/1 ratio, based on comparison of ^13^C NMR spectral data with that reported in the literature [14]. ^13^C NMR (101 MHz, CDCl_3_) δ 148.3, 148.1, 146.1, 145.2, 134.2, 133.0, 127.3, 126.5, 111.7, 111.0, 110.6, 110.4, 74.0, 73.3, 44.8, 44.6, 43.5, 43.1, 32.1, 31.8, 30.8, 30.7, 29.8, 28.4, 23.7, 23.7, 23.1, 23.0, 22.9, 22.6.

Subfractionation of fraction G (57 mg) was accomplished using an XP-Sil, 100 g, SNAP cartridge (40–63 μm, 60 Å, 40 x 150 mm) running at 40 mL min^-1^ using a two step gradient from 50% hexane to 100% DCM over 1911 mL followed by isocratic 100% DCM over 1000 mL. Column eluate was collected into 22 mL portions and based on TLC similarities, recombined into 7 fractions (G-A, 5 mg; G-B, 25 mg; G-C, 3 mg; G-D, 3 mg; G-E, 3 mg; G-F, 6;G-G, 5).

Subfraction G-B was identified as (E)-en-yn-dicycloether based on comparison of ^13^C NMR spectral data with that reported in the literature [15]. ^13^C NMR (101 MHz, CDCl_3_) δ 168.8, 135.8, 125.9, 120.9, 79.9, 79.7, 76.7, 71.5, 69.7, 65.0, 35.5, 24.5, 4.7.

Subfraction G-F was identified as spathulenol by comparison of ^1^H and ^13^C NMR data with an authentic standard that had been isolated in our lab previously [2].

### Insects

As described in detail previously [5], *Aedes aegypti* larvae and adults used in these studies were from a laboratory colony maintained at the Mosquito and Fly Research Unit at the Center for Medical, Agricultural and Veterinary Entomology, USDA-ARS, Gainesville, Florida. For biting deterrence bioassays, eggs were hatched and the insects were reared to the adult stage in the laboratory and maintained at 27 ± 2° C and 60 ± 10% RH with a photoperiod regimen of 12:12 h (L: D). Since this mosquito is active at day time, the bioassays were conducted between 11:00–16:00 hours. 10-18-d-old adult females were used.

### K&D mosquito biting bioassays

As described in detail previously [5], experiments were conducted by using a six-celled *in vitro* Klun and Debboun (K&D) module bioassay system developed for quantitative evaluation of biting deterrent properties of candidate compounds [16]. Briefly the assay system consists of a six-well reservoir with each of the 3 × 4 cm wells containing 6 mL of feeding solution. As described by Ali et al., a feeding solution consisting of CPDA-1 (citrate-phosphate-dextrose-adenine) and ATP was used instead of blood [17]. Green fluorescent tracer dye (www.blacklightword.com) was used to determine feeding by the females. Essential oils, extracts and individual compounds were tested in this study. Essential oil and the extracts were applied at concentrations of 10 μg/cm^2^, pure compounds at 25 nmol/cm^2^, and DEET (97%, *N*, *N*-diethyl-*meta*-toluamide) (Sigma Aldrich, St. Louis, MO) at 25 nmol/cm^2^ as positive control. All treatments were freshly prepared in molecular biology grade 100% ethanol (Fisher Scientific Chemical Co. Fairlawn, NJ) at the time of bioassay. Temperature of the solution in the reservoirs was maintained at 37°C by continuously passing warm water through the reservoir using a circulatory bath. Reservoirs were covered with a layer of collagen membrane (Devro, Sandy Run, SC). Test compounds were randomly applied to six 4 × 5 cm areas of organdy cloth and positioned over the membrane-covered CPDA-1+ATP solution with a Teflon separator placed between the treated cloth and the six-celled module to prevent contamination of the module. A six-celled K&D module containing five female mosquitoes per cell was positioned over the cloth treatments covering the six CPDA-1 + ATP solution membrane wells, and trap doors were opened to expose the treatments to these females. The number of mosquitoes biting through cloth treatments in each cell was recorded after a 3-min exposure and mosquitoes were prodded back into the cells to check the actual feeding. Mosquitoes were squashed and the presence or absence of green fluorescent tracer dye in the gut was used as an indicator of feeding. A replicate consisted of six treatments: four test materials, DEET (a standard biting deterrent) and ethanol-treated organdy as solvent control applied randomly. Four replications each with 5 females per treatment were conducted for the essential oil, extracts and pure compounds.

### *In vitro* A & K repellent bioassay

As described in detail previously [18], the A & K bioassay system is based on the concept that the mosquitoes are attracted to warm temperatures and this system uses warm temperature to serve as stimulus for landing and feeding. The cage contained 200 ± 10, 8-18-d-old female mosquitoes. Numbers of females landing and biting were recorded visually for 1 min. To ensure proper landing and biting, we used 3–4 cages at a time and only one treatment replication of individual compounds was completed in a single cage. As per the criterion, the minimum dose at which feeding was ≤1% was considered as MED. Total 5–10 replicates with one set of mosquitoes were conducted per day.

### Statistical analyses

Proportion not biting (PNB) was calculated using the following formula:

$$PNB=1-\left(\frac{Total\;number\;of\;females\;biting}{Total\;number\;of\;females}\right)$$

PNB values were analyzed using the ANOVA procedure of SAS, and means were separated using the Ryan-Einot-Gabriel-Welsch Multiple Range Test [19].

### Results and discussion

**Fig 1:**
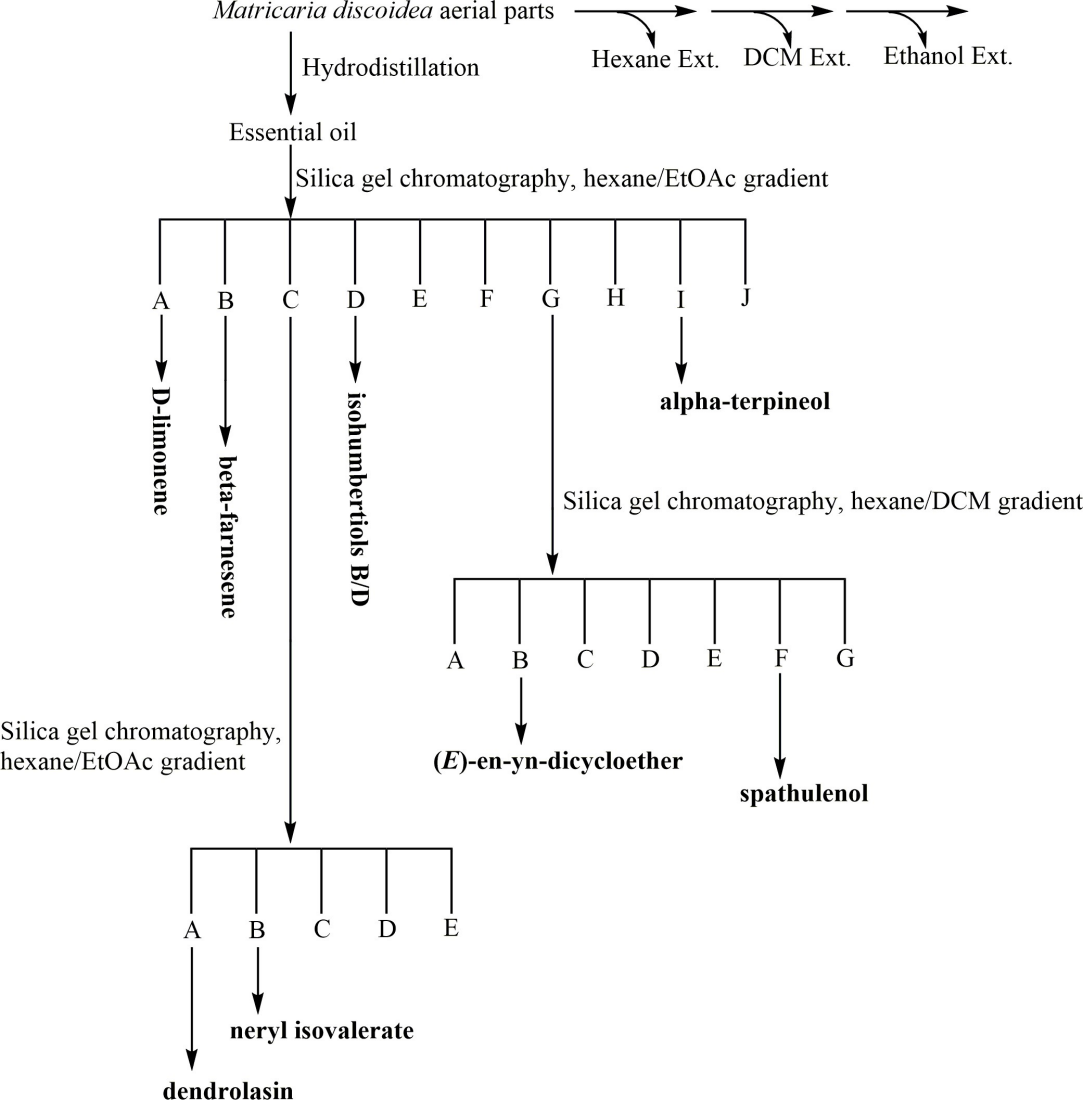
Flow chart depicting the bioassay-directed fractionation pathway used for *M*. *discoidea*.

The areal portion of *M*. *discoidea* plants was collected from the wild and extracted with hexane, methylene chloride, ethanol, and via hydrodistillation. All extracts were evaluated for mosquito biting deterrency against *Ae*. *aegypti* at 10 μg/cm^2^ using an *in-vitro* K&D Module bioassay system and compared against DEET at 4.7 μg/cm^2^ (25 nmoles/cm^2^) and pure ethanol as solvent control. Essential oil produced via hydrodistillation provided a proportion not biting (PNB) value of 0.76 which was statistically equivalent to DEET and more effective than the other extracts (Table 1). Hexane, methylene chloride (DCM), and ethanol extracts demonstrated PNB values of 0.45, 0.52, and 0.47, respectively. None of the solvent based extracts were as effective as DEET or the essential oil, nor were they statistically different from the solvent control. Based on these findings, the essential oil extract was subjected to a bioassay-directed fractionation study (Fig 1).

**Table 1:** Biting deterrent activity of DEET and *M*. *discoidea* extracts against *Ae*. *Aegypti* in K&D bioassays.

Treatment	n^a^	Concentration (μg/cm^2^)	Proportion not biting
**DEET (positive control)**	5	4.8	0.84A
**essential oil (hydrodistillation)**	5	10	0.72AB
**methylene chloride extract**	5	10	0.52BC
**hexane extract**	5	10	0.48C
**ethanol extract**	5	10	0.44C
**ethanol (solvent control)**	5	n/a	0.32C

^a^ n = number of replications with 5 females per replication. Means not followed by the same letter are significantly different (Ryan-Einot-Gabriel-Welsch multiple range test P≤0.05).

Bioactive essential oil from the hydrodistillation was fractionated using silica gel column chromatography (CC) and combined into ten distinct fractions based on their thin layer chromatography (TLC) profile similarity. Fractions were evaluated for mosquito biting deterrent activity against *Ae*. *aegypti* at 10 μg/cm^2^ using the K&D bioassay system and compared against DEET at 4.8 μg/cm^2^ and a solvent control (Table 2).

**Table 2:** Biting deterrent activity of DEET and *M*. *discoidea* fractions against *Ae*. *Aegypti* in K&D bioassays.

Experiment	Treatment	n^a^	Concentration (μg/cm^2^)	Proportion not biting	Standard error
**1**	**ethanol (solvent control)**	5	n/a	0.40	0.04
	**DEET (positive control)**	5	4.8	0.84	0.04
	**essential oil fraction A**	5	10	0.88	0.08
	**essential oil fraction B**	5	10	0.72	0.049
	**essential oil fractions C**	5	10	0.72	0.08
	**essential oil fraction D**	5	10	0.88	0.049
**2**	**ethanol (solvent control)**	5	n/a	0.32	0.049
	**DEET (positive control)**	5	4.8	0.80	0.00
	**essential oil fraction E**	5	10	0.76	0.04
	**essential oil fractions F**	5	10	0.60	0.00
	**essential oil fraction G**	5	10	0.76	0.074
	**essential oil fraction H**	5	10	0.6	0.089
**3**	**ethanol (solvent control)**	5	n/a	0.28	0.08
	**DEET (positive control)**	5	4.8	0.80	0.0
	**essential oil fraction I**	5	10	0.56	0.04
	**essential oil fraction J**	5	10	0.56	0.04

^a^ n: number of replications.

**Fig 2:**
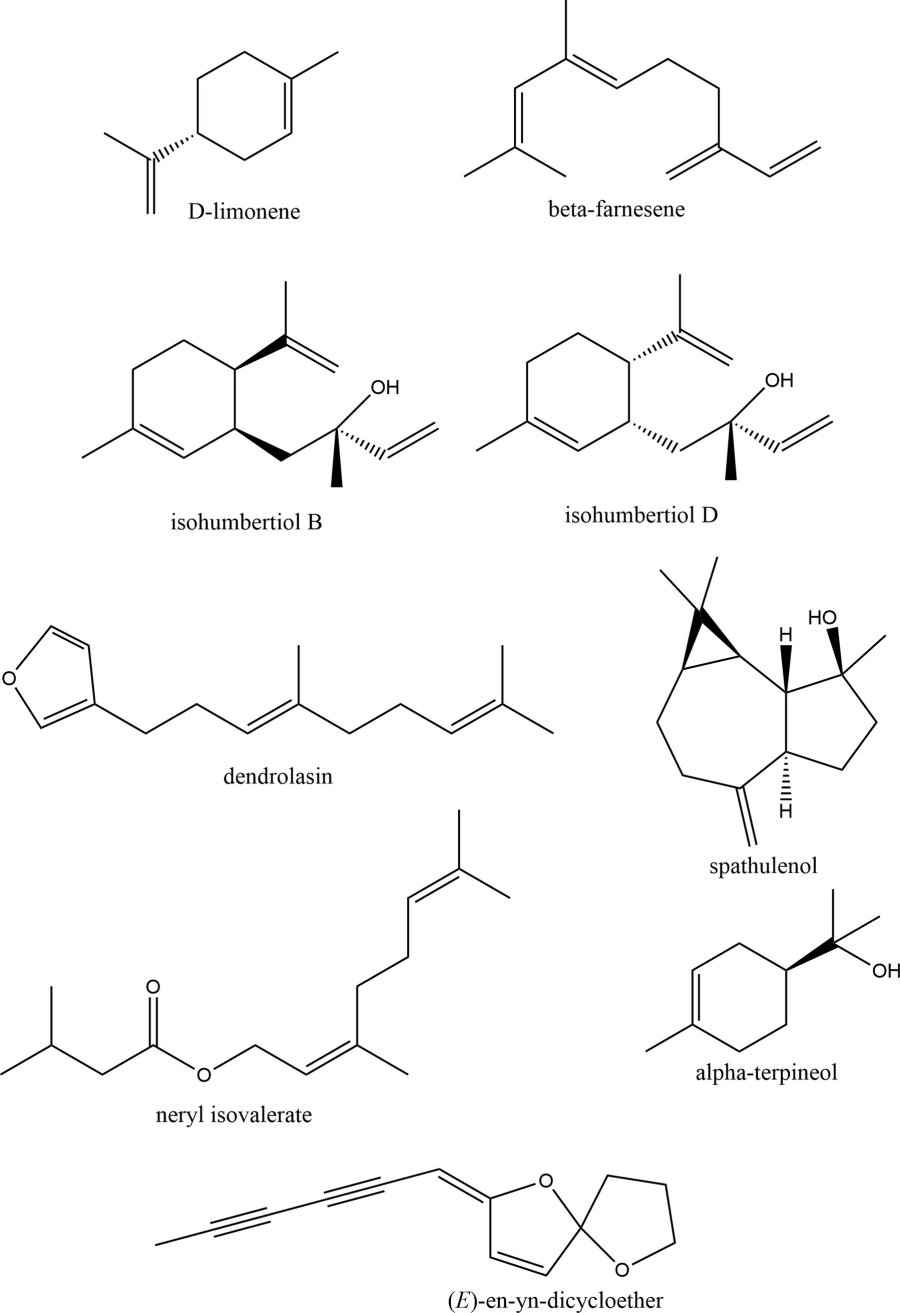
Chemical structure of compounds isolated from *M*. *discoidea* essential oil.

In experiment 1, fractions A to D provided PNB values statistically similar to DEET. In experiment 2, biting deterrent activity of fractions E and G with values of 0.76 were statistically similar to DEET. Fractions F and H with a PNB values of 0.60 were not similar to DEET but were significant above solvent control. In experiment 3, fractions I and J with PNB value of 0.56 showed lower activity than DEET but were significant above solvent control. The most effective fractions were selected for further investigation using chromatographic (CC, GC with retention indices) and spectroscopic techniques (MS, ^1^H and ^13^C NMR). Fractions C and G were subjected to a second round of purification as they contained multiple compounds while fractions A, B, D, and I were pure (Fig 2).

GC/MS analysis of fraction A indicated the presence of one major compound with a strong National Institute of Standards and Technology (NIST) library match for *D*-limonene. The similarity of the calculated RI value (1020) to that reported by Adams [11] and comparison of the GCMS retention time and mass spectrum with an authentic standard unequivocally confirmed the structure as that of *D*-limonene. Commercially available *D*-limonene was used for biological evaluation studies.

Similarly GC/MS analysis of fraction B indicated the presence of one major compound with a strong NIST library match for (*E*)-β-farnesene. The similarity of the calculated RI value (1458) to that reported by Adams [11] and comparison of the GCMS retention time and mass spectrum with an authentic standard unequivocally confirmed the structure as that of (*E*)-β-farnesene.

Further fractionation of the impure fraction C using silica gel column chromatography (CC) provided five subfractions CA to CE. Fraction CA was identified as dendrolasin based on comparison of ^13^C NMR spectral data with that reported in the literature [12]. Subfraction CB was identified as neryl isovalerate based on comparison of ^1^H NMR spectral data with that reported in the literature [13].

Fraction D was identified as isohumbertiols, specifically isohumbertiol B and isohumbertiol D in a 1/1 ratio, based on comparison of ^13^C NMR spectral data with that reported in the literature by Weyerstahl et. al. [14]. Weyerstahl et. al. performed a total synthesis of the stereoisomers of isohumbertiol and also reported the chemical shift as a mixture of inseperable isomers B and D.

Further fractionation of the impure fraction G using silica gel column chromatography (CC) provided 7 subfractions labelled GA to GG. Subfraction G-B was identified as (E)-en-yn-dicycloether based on comparison of ^13^C NMR spectral data with that reported in the literature [15]. Subfraction G-F was identified as spathulenol by comparison of ^1^H and ^13^C NMR data with an authentic standard that had been isolated in our lab previously [2].

**Fig 3:**
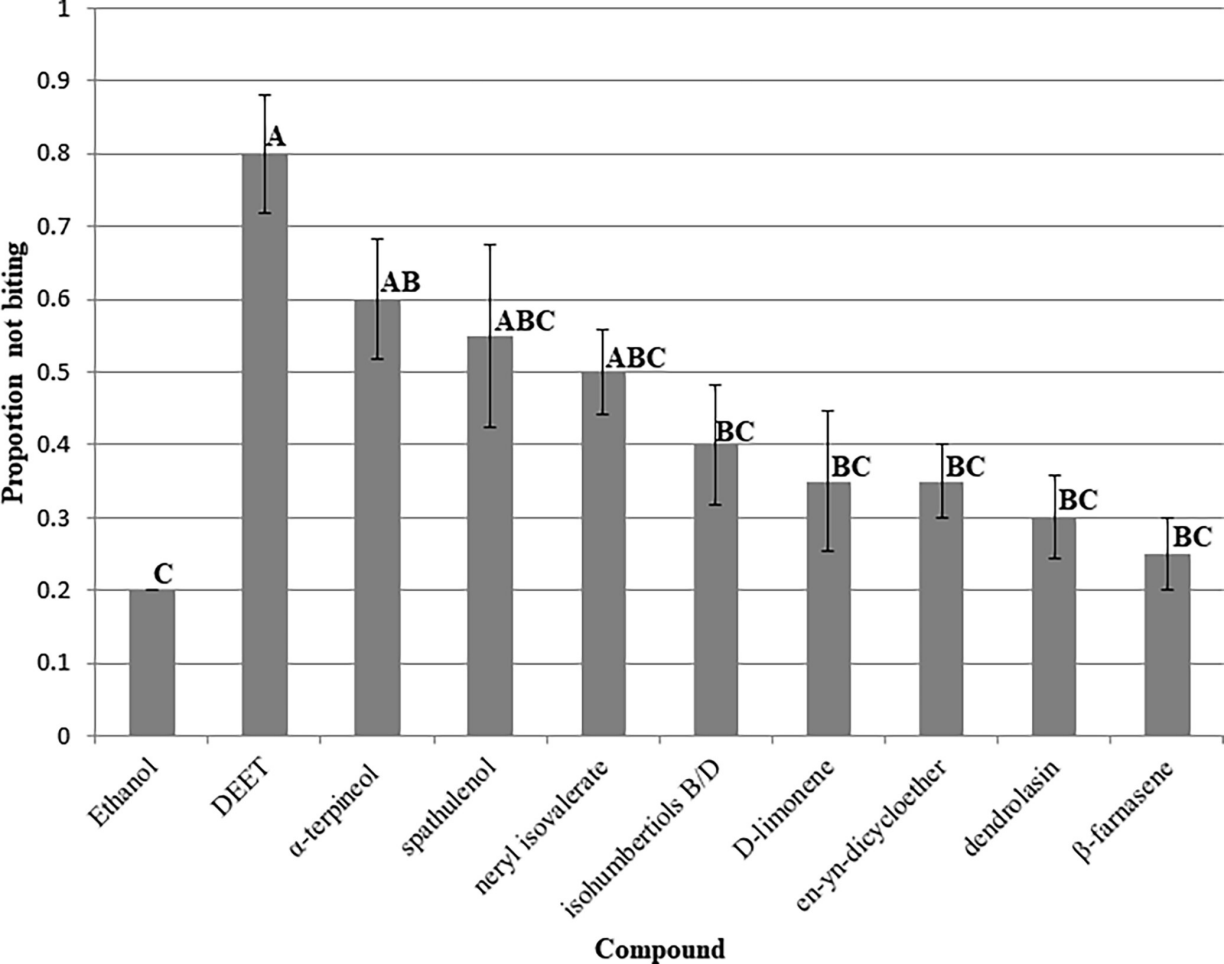
Proportion not biting (± SE) values of pure compounds isolated from *M*. *discoidea* essential oil against female *Ae*. *aegypti*. All compounds tested at 25 nmoles/cm^2^. Ethanol was the solvent control and DEET was used as a positive control. Means not followed by the same letter across columns are significantly different (Ryan-Einot-Gabriel-Welsch multiple range test P≤0.05).

Compounds isolated from active fractions were submitted to mosquito repellency evaluations using the K & D bioassay described above (Fig 3). While crude extracts and fractions are typically evaluated at 10 μg/cm^2^, pure compounds are tested for repellency at 25 nmoles/cm^2^ which was a lower dose than 10 μg/cm^2^ for all compounds tested. A concentration of 25 nmoles/cm^2^ is roughly the lower threshold of DEET in the K & D bioassay to give any bites. Overall all of the compounds isolated from bioactive fractions demonstrated effectiveness against *Ae*. *aegypti* except for β-farnasene. β-farnasene was not significantly active above ethanol control. None of the compounds were as effective as DEET at 25 nmoles/cm^2^. α-terpineol had the highest PNB of (0.60 ± 0.08) compared to that of DEET at 0.80 ± 0.08. This was followed by spathulenol and neryl isovalerate with PNB values of 0.55 ± 0.13 and 0.50 ± 0.06, respectively, both of which were statistically similar to α-terpineol. Isohumbertiols B/D, D-limonene, en-yn-dicycloether, and dendrolasin were all statistically equivalent and had PDB values ranging between 0.40 to 0.30.

These same isolated compounds were submitted to mosquito repellency evaluations against *Ae*. *aegypti* using the A & K *in vitro* bioassay (Table 3) [18]. Compounds were evaluated at concentrations of 50, 25, and 12.5 μg/cm^2^ and DEET was used as a positive control. α-terpineol demonstrated complete repellency at 50 and 25 μg/cm^2^ but failed at 12.5 μg/cm^2^.

**Table 3:** Repellent activity of DEET and pure compounds isolated from *M*. *discoidea* essential oil against female *Ae*. *aegypti* in A & K bioassays.

	Percentage of females biting out of 200 in the cage^a^		
	50 μg/cm^2^	25 μg/cm^2^	12.5 μg/cm^2^
**DEET**	0.0 ± 0.0	0.0 ± 0.0	0.3 ± 0.05
**Isohumbertiol B/D**	>1	>1	>1
***D*-limonene**	>1	>1	>1
***β*-farnesene**	>1	>1	>1
***α*-terpineol**	0	0	>1
**(*E*)-en-yn-dicycloether**	>1	>1	>1
**spathulenol**	>1	>1	>1
**dendrolasin**	>1	>1	>1
**neryl isovalerate**	>1	>1	>1

^a^Data are %age (mean ± SEM) biting; Minimum effective dose (MED) is ≤ 1% biting which are ≤ 2 females out of 200.

It is very common in the literature to see reports on essential oils with mosquito repellent activity without a corresponding bioassay-directed fractionation of the oils to specifically attribute the mosquito repellent activity of individual compounds. Often the oil activity is reported only and we are left to speculate on which individual constituents are responsible for the activity of the oil as a whole. Many of these essential oil evaluations are reported to contain some of the constituents isolated from this study that likely contribute to their efficacy; however, there is no evidence in these manuscripts that identify the specific constituents are responsible for the activity.

There are thorough evaluations of individual compounds for a few of the constituents isolated in this study. For example, limonene was previously isolated from *Artemisia vulgaris* and found to work as a repellent against *Ae*. *aegypti* at 1.4 mg/cm^2^ [20]. α-terpineol has been reported from a variety of natural sources and most recently, it was shown to possess a MED value of 0.039 mg/cm^2^ in human evaluations for topical repellency [21]. Spathulenol was reported to be partly responsible for the *Ae*. *aegypti* repellency of the traditionally used plant *Callicarpa americana* [2]. In this report, spathulenol demonstrated a PNB of 0.73 at 25 nmoles/cm^2^ as compared to the positive control SS220 with a PNB of 0.80 at 25 nmoles/cm^2^. This value is similar to that which we observed in this study. Its worth noting that in this investigation and the previous investigation spathulenol was observed by NMR to degrade quickly, making it difficult to obtain repellency data before some decomposition occurs. Furthermore, when working with small quantities of spathulenol and the requirement to remove solvent via evaporation during purification, combined with the high volatility of spathulenol, all make it difficult to perform such repellency bioassays due to the difficulty in obtaining an accurate weight of the sample for bioassay purposes. Hence, the unstable nature and volatility of spathulenol may explain the minor differences observed. This appears to be the first report on the *Ae aegypti* mosquito repellency of isohumbertiols, dendrolasin, neryl isovalerate, and (E)-en-yn-dicycloether. Scientific evidence reported here validates the traditional use of *M*. *discoidea* as a biting-insect deterrent and identified a variety of compounds responsible for this effect. As this is the first report on the insect repellency of neryl isovalerate, more work is needed to determine if this compound can be used as a natural alternative to the more widely used synthetic insect repellents, DEET, IR3535 or picaridin [22].
